# Computational framework of cobalt ferrite and silver-based hybrid nanofluid over a rotating disk and cone: a comparative study

**DOI:** 10.1038/s41598-023-32360-7

**Published:** 2023-04-01

**Authors:** Umar Farooq, Hassan Waqas, Nahid Fatima, Muhammad Imran, Sobia Noreen, Abdul Bariq, Ali Akgül, Ahmed M. Galal

**Affiliations:** 1 Department of Mathematics, Government College University, Faisalabad, 38000 Pakistan; 2 School of Energy and Power Engineering, Jiangsu University, Zhenjiang, 2122013 China; 3 Department of Mathematics and Sciences, Prince Sultan University, Riyadh, 11586 Saudi Arabia; 4 Department of Chemistry, Government College Women University, Faisalabad, 38000 Pakistan; 5 Department of Mathematics, Laghman University, Mehtarlam, 2701 Laghman Afghanistan; 6 Department of Computer Science and Mathematics, Lebanese American University, Beirut, Lebanon; 7 Department of Mathematics, Art and Science Faculty, Siirt University, 56100 Siirt, Turkey; 8 Department of Mathematics, Mathematics Research Center, Near East University, Near East Boulevard, 99138 Nicosia/Mersin 10, Turkey; 9 Department of Mechanical Engineering, College of Engineering in WadiAlddawasir, Prince Sattam Bin Abdulaziz University, Al-Kharj, Saudi Arabia; 10 Production Engineering and Mechanical Design Department, Faculty of Engineering, Mansoura University, Mansoura, P.O 35516 Egypt

**Keywords:** Engineering, Mathematics and computing, Physics

## Abstract

The dominant characteristics of hybrid nanofluids, including rapid heat transfer rates, superior electrical and thermal conductivity, and low cost, have effectively piqued the interest of global researchers. The current study will look at the impacts of a silver and cobalt ferrite-based hybrid nanofluid with MHD between a revolving disk and cone. The collection of partial differentiable equations is converted into a set of ODEs via similarity transformations. We used the Homotopy analysis approach from the BVPh 2.0 package to solve the ordinary differential equations. The volume proportion of nanoparticles increases and the temperature distribution profile also increased. It is more efficient for metallurgical, medicinal, and electrical applications. Furthermore, the antibacterial capabilities of silver nanoparticles might be used to restrict the growth of bacteria. A circulating disc with a stationary cone has been identified to provide the optimal cooling of the cone disc device while maintaining the outer edge temperature constant. This study's findings might be useful in materials science and engineering. The usage of hybrid nanofluid in heat transfer and heat pumps, coolants in manufacturing and production, producing cooling, refrigerators, solar thermal collectors, and heating, air conditioning, and climate control applications are only a few examples.

## Introduction

Important expansion has been developed in the variety of science and the vast range of demands for practical nanotechnology in current decades. Nanoparticles have a wide range of possible uses, like biomedical, biotechnology, crystal chemistry, statistical modeling, and disciplines such as sociology, petroleum neuroscience, and so on. Surface textures, shapes, and proportions are some of the additional physical properties of nanoparticles that must be measured for an accurate representation. Nanoparticles are used in a broad range of interdisciplinary fields such as pharmaceutical products, thermal devices, servers, nuclear reactors, chemical plants, and so on. CNTs can also be used in paint, polymer, and indirect interaction with a mucous membrane. In general, nanomaterials technologies focus on increasing the proficiency, durability, and swiftness of existing processes. Nanofluids are legislature fluids made up of micrometers particles. They have special physical properties and engineered carbon nanotubes that can be used in several fields, namely chemical processes, safety, environmental, chemistry, and manufacturing, due to their semi-nanoscopic size. Within the definition, nanomaterials must specifically be classified as such within a range of 1–100 nm under one of their consistency ranges, although their other measurements fall outside of that range. Choi^1^ was recognized as the first to discuss the idea of nanofluids. Buongiorno^2^ identified thermophoresis results and Brownian motion as critical factors that influence the capability of materials production to transmit temperature. Katiyar et al.^3^ examined the basic dynamic effectiveness technique for fluid motion in porous media. In a virtual sample with a temperature gradient, Hayat et al.^4^ examined the stream of fluid (Carreau fluid). Tlili et al.^5^ expressed the flow of Maxwell nanofluid in the reality of heat distribution and generative influences. Maxwell fluid flow through vertical surfaces with thermal flux open heat transfer was studied by Shah et al.^6^. The expanded cylinder was used by Sohail et al.^7^ to understand the measure of Sutterby fluid. The Darcy-Forchheimer heat property of MHD hybrid nanomaterials flow due to strained cylinders was investigated by Saeed et al.^8^. Babazadeh et al.^9^ developed a hypothetical model for nanomaterials migration on the inside of a porous vacuum. Ullah et al.^10^ simulated the heat exchange in a copper-oxidized water-based circular cylinder that was partially heated. The consequences of nanofluid on motile bacteria and Wu's slip were explored by Li et al.^11^. Waqas et al.^12^ investigated the influence of melting on nanofluid flow through a stretched cylinder. Rashid et al.^13^ computed the magnetic flux-related mobility of nanofluids based on adsorption. Xian et al.^14^ discovered a steady nanofluid developed by displacing titanium dioxide (TiO2) and cellulose nanocrystals in a two-step process of purified water. Rabbi et al.^15^ examined the physical fields of convective heat in a steel column of Cu-H2O non-material for various thermostat-sink combinations using the convolution neural network technique as an operative predictive method. Ghalambaz et al.^16^ studied the results of conjugate energy transfer in a horizontal pipe of a novel nanofluid flow (Ag–MgO/water hybrid nanofluid). Huminic and Huminic^17^ investigated the features of nanofluid and hybrid nanofluid processing in different thermal methods for different boundary climates and health conditions. Tayebi^18^ examined the impact of entropy production caused by natural temperature distribution in a tunnel leading between parallel fluorescence microscopy exercise bike cylinders. The effect of different detergents and ultraviolet times on the stabilization and biomedical applications of hybrid supernatant was measured by Xian et al.^19^. Awais et al.^20^ studied the influence of Magnetohydrodynamics on flow adhesives using mechanical and tribological hybrid nanofluid. Waini et al.^21^ explored the MHD convective flow and temperature change of a hybrid nanofluid passing over a porous stretching wedge. Farooq et al.^22^ evaluated the impact of Carreau nanofluid bioconvection flow using modified Cattaneo-Christov expressions. Reddy et al.^23^ examined the consequence of nanofluid flow via square cavity using entropy generation and heat transfer measurements. Reddy et al.^24^ studied the heat transmission effects of a hybrid nanofluid flow from inside a container. Sreedevi and Reddy^25^ evaluated the effects of heat transmission and entropy production analyses on a hybrid nanofluid within a cavity. Sudarsana and Sreedevi^26^ explored the impact of heat transfer analyses on the nanofluid within a cylinder. Sreedevi and Reddy^25^ investigated the thermal transmission and entropy formation of a nanoliquid via a cavity. Reddy et al.^27^studied the impact of MHD flow heat and mass transportation features of a nanofluid via a cone. Reddy et al.^28^ inspected the impact of hybrid nanoliquid characteristics on a slip-effecting sheet. Dero et al.^29^ investigated hybrid nanofluids' impact on suction/injection applications. Haq et al.^30^ explored the surface effects of a radiative viscous hybrid nanofluid based on theoretical research. The effects of curved radiated surfaces on a modified hybrid nanofluid model were examined by Abbasi et al.^31^. Hassan et al.^32^ looked at how heat and mass transfer with hybrid nanofluids. The consequences of hybrid nanofluid flow on a curved stretched sheet were investigated by Madhukesh et al.^33^. Reddy and Sreedevi^34^ studied the influence of heat and mass transmission as well as entropy formation in a hybrid nanofluid. More work on nanofluid and hybrid nanofluid are carried out^12,35–40^.

Current research work aims to analyze the comparative investigation of Cobalt ferrite (COFe_2_O_4_) nanofluid and Silver (Ag), Cobalt ferrite (COFe_2_O_4_) hybrid nanofluid flow over a disk and cone. In this study, we looked at the three-dimensional flow of hybrid nanofluid and nanofluid. It has been demonstrated that hybrid nanofluids greatly increase the thermal efficiency of basic fluids when contrasted to other fluids. The nonlinear problem was evaluated using the Homotopy analysis approach, methodology, and BVPh 2.0 software, and this method was compared to the numerical (ND-solve) method.We used the computational tool MATHEMATICA to compute the graphical flow patron of the flow parameter. Silver nanoparticles are used to modulate a variety of actions, including antibacterial, antifouling, chemotherapeutic, antiviral, and drug-delivery systems. This research work will be an excellent improvement on the existing research on the flow of nanofluids across cone and disk. Silver nanoparticles are increasingly being used in a range of industries due to their unique physical and chemical properties, including medicine, food, socialized medicine, consumer electronics, and industrial. Among them include optical, electrical, and thermal properties, as well as high electrical conductivity and biological properties. Because of its features, cobalt ferrites have been widely used in sensors, recording devices, magnetic cards, solar panels, magnetic medication delivery, pharmaceuticals, catalysis, and biotechnology.

## Physical description and modeling

**Figure 1:**
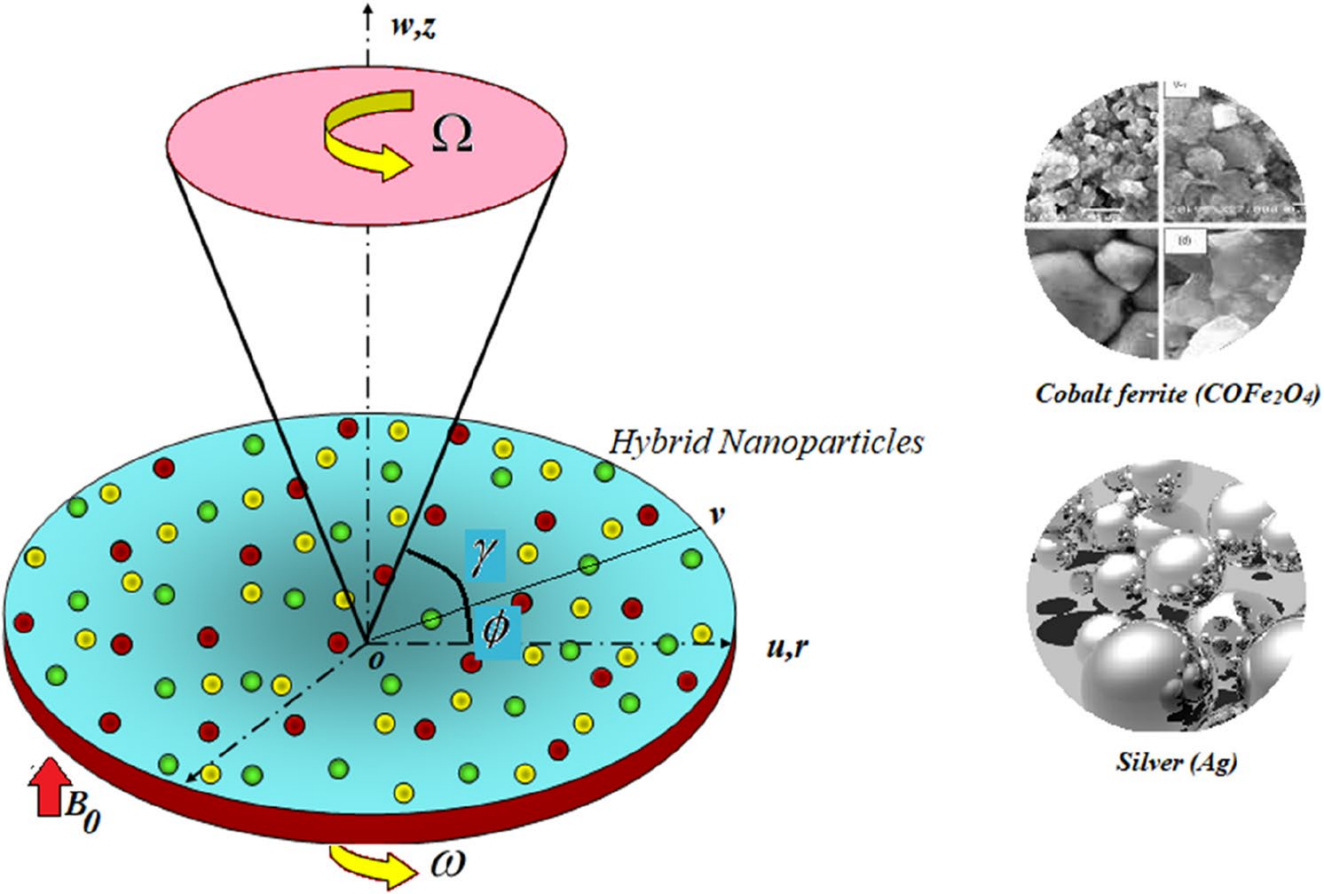
Flow geometry of the problem.

Here is the incompressible flow of hybrid nanoparticles and based fluid over both the geometry disk and cone. Here $\left( {r,\phi ,z} \right)$ are the cylindrical coordinate of the cone & disk and $\left( {B_{0} } \right)$ magnetic field assistance in the direction z, the induced magnetic field is neglected. Here the hybrid nanoparticles*Ag* + COFe_2_O_4_and COFe_2_O_4_ based fluid is used. The angular velocities of the pipe and disk are denoted $\left( {\omega \& \Omega } \right)$ individually. Figure 1 depicts the flow process.

The main equations are^41,42^:

1 $$u_{r} + w_{z} + \frac{u}{r} = 0$$

2 $$\rho_{hnf} \left[ {uu_{r} + wu_{z} - \frac{{v^{2} }}{r}} \right] = - p_{r} + \mu_{hnf} \left[ {u_{rr} + u_{zz} + \frac{1}{r}u_{r} - \frac{v}{{r^{2} }}} \right] - \sigma_{hnf} B_{0}^{2} u,$$

3 $$\rho_{hnf} \left[ {uv_{r} + wv_{z} - \frac{uv}{r}} \right] = \mu_{hnf} \left[ {v_{rr} + v_{zz} + \frac{1}{r}v_{r} - \frac{v}{{r^{2} }}} \right] - \sigma_{hnf} B_{0}^{2} v,$$

4 $$\rho_{hnf} \left[ {uw_{r} + ww_{z} } \right] = - p_{z} + \mu_{hnf} \left[ {w_{rr} + w_{zz} + \frac{1}{r}w_{r} } \right],$$

5 $$(\rho cp)_{hnf} \left[ {uT_{r} + wT_{z} } \right] = k_{hnf} T_{zz} + \sigma_{hnf} B_{0}^{2} (u^{2} + v^{2} ),$$

where $\left( {u,v,w} \right)$ components of speed, While $\left( {\sigma_{hnf} } \right)$ is the electrical conduction, $\left( {B_{0} } \right)$ is the magnetic strength, $\left( {(\rho c_{p} )_{hnf} } \right)$ heat capacitance, $\left( p \right)$ the pressure of the fluid, $\left( {\mu_{hnf} } \right)$ dynamic viscosity, $\left( {\rho_{hnf} } \right)$ density, and $\left( {\,k_{hnf} } \right)$ thermal conductivity respectively.

Boundary conditions^41,42^:

6 $$\left. \begin{gathered} \left[ {u\left( { = 0} \right)} \right],\,\,\,\,\,\,\,\,\,\,\,\left[ {w\left( { = 0} \right)} \right],\,\,\,\,\,\left[ {T\left( { = T_{w} } \right)} \right],\,\,\,\,\,\,\left[ {v\left( { = \omega r} \right)} \right], \hfill \\ \left[ {z\left( { = 0} \right)} \right] \hfill \\ \left( {u\left( { = 0} \right)} \right),\,\,\,\,\,\,\,\,\,\,\,\left[ {w\left( { = 0} \right)} \right],\,\,\,\,\,\left[ {T\left( { = T_{\infty } } \right)} \right],\,\,\,\,\,\,\left[ {v\left( { = \Omega r} \right)} \right], \hfill \\ \left[ {z\left( { = r\tan \gamma } \right)} \right], \hfill \\ \end{gathered} \right\},$$

Here $\gamma$ show the angle between the disk and the cone.

Similarity transformations^41,42^:

7 $$\left. \begin{gathered} \left[ {U_{w} f(\zeta )} \right] = u = \left[ {\frac{{\upsilon_{f} f(\zeta )}}{r}} \right],v = \left[ {\frac{{g(\zeta )\upsilon_{f} }}{r}} \right] = \left[ {U_{w} g(\zeta )} \right],\zeta = \left[ \frac{z}{r} \right],\, \hfill \\ w = \left[ {\frac{{\upsilon_{f} h(\zeta )}}{r}} \right] = \left[ {U_{w} h(\zeta )} \right],\,p = \left[ {\frac{{\rho \upsilon_{f}^{2} P}}{{r^{2} }}U_{w}^{2} \rho .p} \right],\theta = \left[ {\frac{{T - T_{\infty } }}{{T_{w} - T_{\infty } }}} \right]\, \hfill \\ \end{gathered} \right\},$$

Here $M = \left( {\frac{{v_{f} \sigma_{f} B_{0}^{2} }}{{\rho_{f} U_{w}^{2} }}} \right)$ the magnetic parameter, $\left( {U_{w} } \right)$ is the surface velocity, and $\Pr = \left( {\frac{{\mu_{f} Cp}}{{k_{f} }}} \right)$ the Prandtl number.

Now, by using (7) the model of Eq. (2–6) is as:

8 $$\left. {h^{\prime} - \zeta f^{\prime}} \right\} = 0,$$

9 $$\left. \begin{gathered} 3\zeta f^{\prime} + (1 + \zeta^{2} )f^{\prime\prime} + (1 - \phi_{{CoFe_{2} O_{4} }} )^{2.5} (1 - \phi_{Ag} )^{2.5} \hfill \\ \times \left[ {\zeta ff^{\prime} - hf^{\prime} + f^{2} - g^{2} } \right] \hfill \\ \left[ {(1 - \phi_{Ag} )\left( {1 - \left( {1 - \frac{{\rho_{{CoFe_{2} O_{4} }} }}{{\rho_{f} }}} \right)\phi_{{CoFe_{2} O_{4} }} } \right) + \phi_{Ag} \left( {\frac{{\rho_{Ag} }}{{\rho_{f} }}} \right)} \right] \hfill \\ + (1 - \phi_{{CoFe_{2} O_{4} }} )^{2.5} (1 - \phi_{Ag} )^{2.5} [2p - MF + \zeta p^{\prime}] \hfill \\ \end{gathered} \right\} = 0,$$

10 $$\left. \begin{gathered} g^{\prime\prime}(1 + \zeta^{2} ) + g^{\prime}3\zeta - (1 - \phi_{{CoFe_{2} O_{4} }} )^{2.5} (1 - \phi_{Ag} )^{2.5} \left[ {\zeta fg^{\prime} - hg^{\prime}} \right] \hfill \\ \left[ {(1 - \phi_{Ag} )\left( \begin{gathered} 1 - \hfill \\ \left( {1 - \frac{{\rho_{{CoFe_{2} O_{4} }} }}{{\rho_{f} }}} \right)\phi_{{CoFe_{2} O_{4} }} \hfill \\ \end{gathered} \right) + \phi_{Ag} \left( {\frac{{\rho_{Ag} }}{{\rho_{f} }}} \right)} \right] - (1 - \phi_{{CoFe_{2} O_{4} }} )^{2.5} Mg(1 - \phi_{Ag} )^{2.5} = 0 \hfill \\ \end{gathered} \right\},$$

11 $$\left. \begin{gathered} (1 + \zeta^{2} )h^{\prime\prime} + 3\zeta h^{\prime} + (1 - \phi_{{CoFe_{2} O_{4} }} )^{2.5} (1 - \phi_{Ag} )^{2.5} \times \left[ d \right]\left[ { - hh^{\prime} + h + \zeta fh^{\prime} + fh} \right] \hfill \\ - (1 - \phi_{{CoFe_{2} O_{4} }} )^{2.5} (1 - \phi_{Ag} )^{2.5} p^{\prime} = 0 \hfill \\ \end{gathered} \right\},$$

12 $$\left. \begin{gathered} \frac{{k_{hnf} }}{{k_{nf} }}\left[ {\zeta (1 - 2n)\theta \prime + n^{2} \theta + (1 + \zeta^{2} )\theta \prime \prime } \right] + \left[ {\zeta f\theta \prime - nf\theta - h\theta \prime } \right] \hfill \\ \Pr \left[ {(1 - \phi_{Ag} )\left( {1 - \left( {1 - \frac{{(\rho C_{p} )_{{CoFe_{2} O_{4} }} }}{{(C_{p} \rho )_{f} }}\phi_{{CoFe_{2} O_{4} }} } \right) + \frac{{(\rho C_{p} )\phi_{Ag} }}{{(C_{p} \rho )_{f} }}\phi_{Ag} } \right)} \right] \hfill \\ + \frac{M}{{(1 - \phi_{Ag} )^{2.5} (1 - \phi_{{CoFe_{2} O_{4} }} )^{2.5} }}(f^{2} + g^{2} ) = 0 \hfill \\ \end{gathered} \right\},$$

Boundary conditions:

13 $$\left. \begin{gathered} f(0)\left( { = 0} \right),\,\,\,\,\,\,\,\,\,\,\,\,\,\,\theta (0)\left( { = 1} \right),\,\,\,\,\,\,\,\,\,\,\,\,\,\,\,\,\,\,h(0)\left( { = 0} \right), \hfill \\ g(0)\left( { = Re_{\omega } } \right),\,\,\,\,\,\,\,\,\,\,\,\,\,\,\,\,\,\,\,\,\,\,\,\,\,\,\,\,\,\,\,\,\,\,\,\,\,\,\,g(\zeta_{0} )\left( { = {\text{Re}}_{\Omega } } \right), \hfill \\ f(\zeta_{0} )\left( { = 0} \right),\,\,\,\,\,\,\,\,\,\,\,\,(\zeta_{0} )\left( { = 0} \right),\,\,\,\,\,\,\,\,\,\,\,\,\,\,\,\,\,h(\zeta_{0} )\left( { = \,0} \right) \hfill \\ \end{gathered} \right\},$$

The volume fraction of nanoparticles is $\phi_{{COFe_{2} O_{4} }} \,and\,\phi_{Ag}$.

Here $\left( {\sigma_{hnf} } \right)$ is electrical conduction, $\left( {\rho_{hnf} } \right)$ density, $\left( {v_{hnf} } \right)$ kinematic viscosity, $\left( {k_{hnf} } \right)$ thermal conductivity, and $\left( {Cp_{hnf} } \right)$ certain heat.

14 $$\left. \begin{gathered} Nu_{d} = - \frac{{k_{hnf} }}{{k_{nf} }}\theta^{\prime}(0), \hfill \\ Nu_{c} = - \frac{{k_{hnf} }}{{k_{nf} }}\theta^{\prime}(\zeta_{0} ) \hfill \\ \end{gathered} \right\}.$$

Here $Nu_{c}$ is the Nusselt number for the cone and $Nu_{d}$ the disk.

## Numerical procedure

In this article, we have used HAM to solve the modeled equations. Liao's HAM approach solves all high solutions by a sufficient choice of model parameters to enable a divergent sequence solution. In mathematical approaches, HAM can solve boundary value problems. In contrast to perturbation systems, HAM solutions do not require the collection of small/large parameters. Rather than the physical quantity, the auxiliary parameter controls the convergence of the sequence solutions. HAM also gives us the freedom to use our first-guess calculations while having the flow system. The amount of residual error is estimated using the BVP 2.0 to show the convergence speed. This approach selects preliminary estimations that the boundary conditions. To run the MATHEMATICA tool using the HAM technique, initial guesses are needed.

15 $$\left. \begin{gathered} f_{0} (\zeta )\left( { = 0} \right),\,\,\,\,\,\,\,\,\,\,g_{0} (\zeta )\left( { = \frac{{(Re_{\Omega } - {\text{Re}}_{\omega } )}}{{\zeta_{0} }}\zeta + {\text{Re}}_{w} } \right), \hfill \\ h_{0} (\zeta )\left( { = 0} \right),\,\,\,\,\,\,\,\,\,\,\,\,\,\,\,\,\,\,\,\,\,\,\,\,\,\,\,\,\,\,\,\,\,\,\,\,\,\,\,\,\theta_{0} (\zeta )\left( { = \frac{{\zeta_{0} - \zeta }}{{\zeta_{0} }}} \right) \hfill \\ \end{gathered} \right\},$$

So,

16 $$\ell_{h} (h) = h^{\prime\prime},\ell_{\theta } (\theta ) = \theta^{\prime\prime},\ell_{f} (f) = f^{\prime\prime},\ell_{g} (g) = g^{\prime\prime}$$

The expanded form

17 $$\ell_{f} [\chi_{1} + \chi_{2} \zeta ] = 0,\ell_{g} [\chi_{3} + \chi_{4} \zeta ] = 0,\ell_{h} [\chi_{5} + \chi_{6} \zeta ] = 0,\ell_{\theta } [\chi_{7} + \chi_{8} \zeta ] = 0.$$

From Eqs. (09–12) as

18 $$\varepsilon_{m}^{f} = \left( {\frac{1}{n + 1}} \right)\,\sum\limits_{x = 1}^{n} {\left[ {N_{f} \left( {\left( {\sum\limits_{y = 1}^{m} {f(\zeta ),\,} } \right)\left( {\sum\limits_{y = 1}^{m} {g(\zeta )} } \right),\left( {\sum\limits_{y = 1}^{m} {h(\zeta )} } \right)} \right)_{\zeta = x\delta \zeta } } \right]^{2} } ,$$

19 $$\varepsilon_{m}^{g} = \left( {\frac{1}{n + 1}} \right)\sum\limits_{x = 1}^{n} {\left[ {N_{g} \left( {\left( {\sum\limits_{y = 1}^{m} {h(\zeta )} } \right),\left( {\sum\limits_{y = 1}^{m} {f(\zeta )} } \right),\left( {\sum\limits_{y = 1}^{m} {g(\zeta )} } \right)} \right)_{\zeta = x\delta \zeta } } \right]^{2} } ,$$

20 $$\varepsilon_{m}^{h} = \left( {\frac{1}{n + 1}} \right)\,\sum\limits_{x = 1}^{n} {\left[ {N_{f} \left( {\,\sum\limits_{y = 1}^{m} {h(\zeta )} } \right)_{\zeta = x\delta \zeta } } \right]^{2} } ,$$

21 $$\varepsilon_{m}^{\theta } = \left( {\frac{1}{n + 1}\,} \right)\,\sum\limits_{x = 1}^{n} {\left[ {N_{\theta } \left( {\left( {\sum\limits_{y = 1}^{m} {f(\zeta )} } \right),\left( {\sum\limits_{y = 1}^{m} {\theta (\zeta )} } \right),\left( {\sum\limits_{y = 1}^{m} {g(\zeta )} } \right)} \right)_{\zeta = x\delta \zeta } } \right]^{2} } ,$$

22 $$\varepsilon_{m}^{t} = \varepsilon_{m}^{f} + \varepsilon_{m}^{g} + \varepsilon_{m}^{h} + \varepsilon_{m}^{\theta } .$$

## Results and discussion

**Figure 2:**
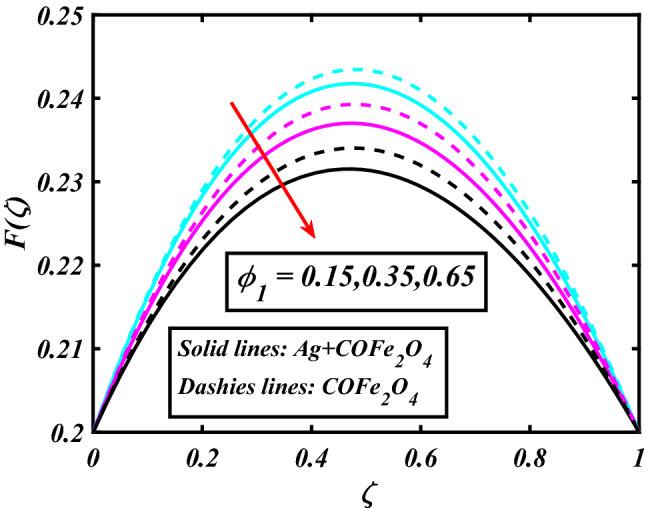
Demonstration of $\phi_{1}$ through $F\left( \zeta \right)$.

**Figure 3:**
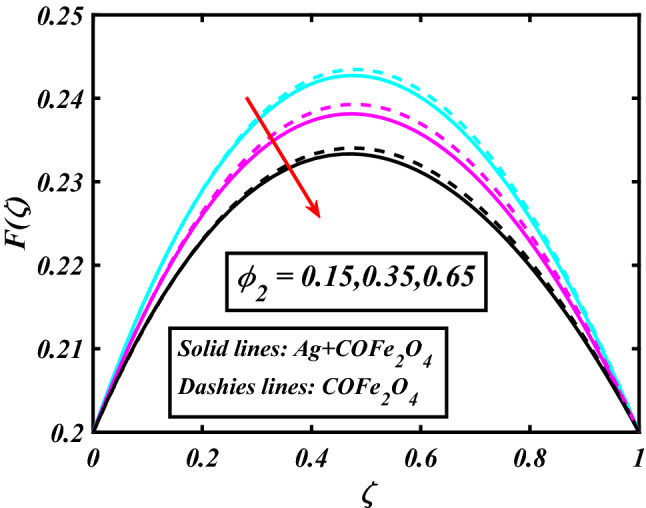
Demonstration of $\phi_{2}$ through $F\left( \zeta \right)$.

**Figure 4:**
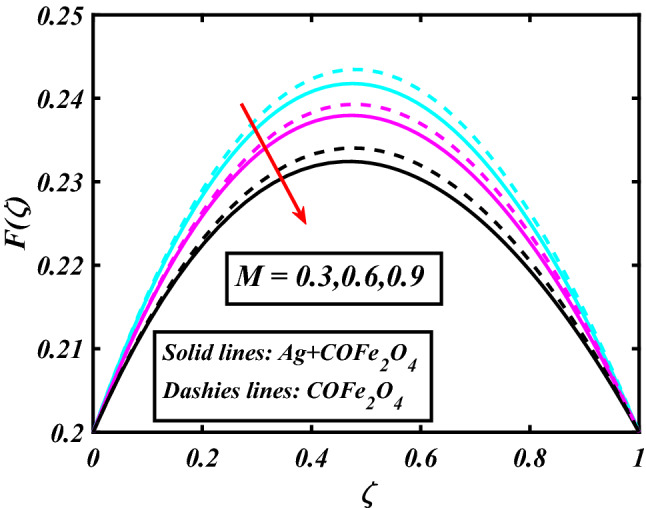
Demonstration of $M$ through $F\left( \zeta \right)$.

**Figure 5:**
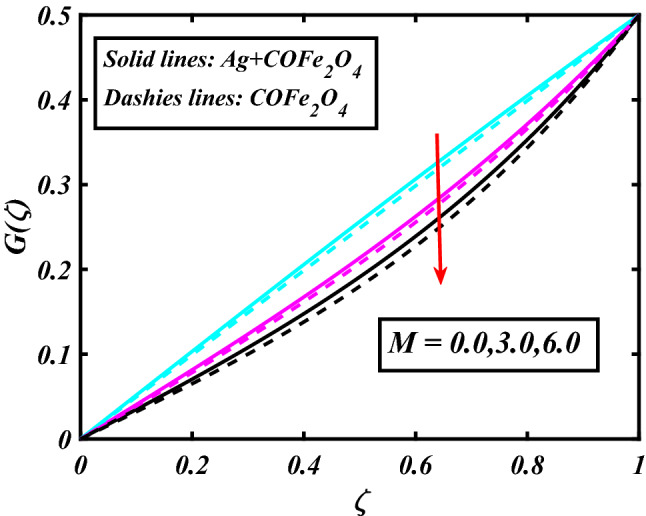
Demonstration of $M$ through $G\left( \zeta \right)$.

**Figure 6:**
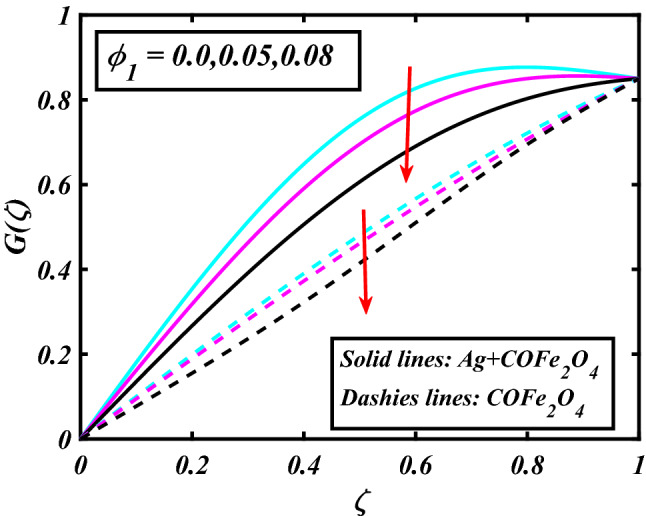
Demonstration of $\phi_{1}$ through $G\left( \zeta \right)$.

**Figure 7:**
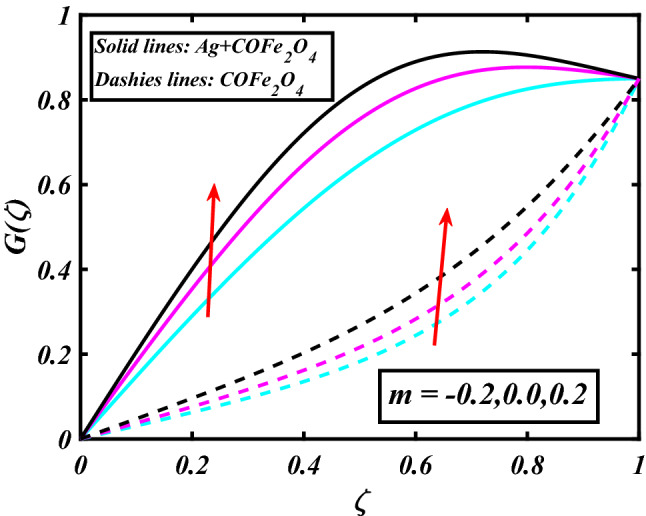
Demonstration of $m$ through $G\left( \zeta \right)$.

**Figure 8:**
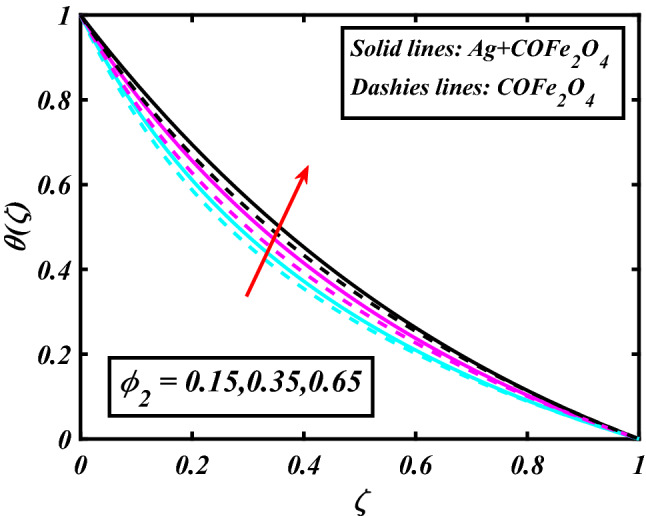
Demonstration of $\phi_{2}$ through $\theta \left( \zeta \right)$.

**Figure 9:**
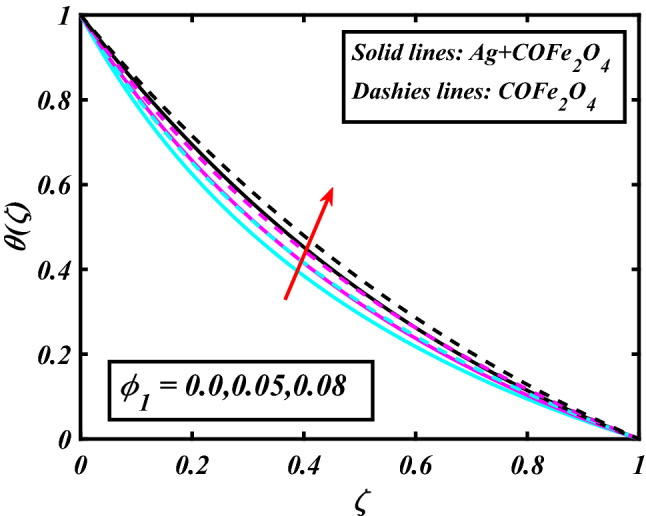
Demonstration of $\phi_{1}$ through $\theta \left( \zeta \right)$.

The system (09–12) is numerically solved by the Homotopy analysis technique. Noticeable performances of the interesting constraints on velocity and temperature are graphically investigated. By taking nonlinear flow parameters of nanofluid and hybrid nanofluid flow traveling through a rotating disk and cone are addressed. To solve the model's non-linear boundary value problem, the ND-solve approach in the MATHEMATICA tool using the HAM technique is employed. This method is used to address the boundary value issue of an ordinary differential comparison. This section's main goal is to define and expound on the dimensionless parametric impact on flow velocity and temperature. To test the accuracy of the suggested model, we repeated the method with a wide range of parameter values. The values of these parameters have a significant impact on the convergence of these series. The performance of the volume amended of nanoparticles $\left( {\phi_{1} } \right)$ on the velocity distribution profile $F\left( \zeta \right)$ is noticed in Fig. 2. It can be understood that the velocity distribution profile $F\left( \zeta \right)$ has declining performance for distension estimates of the capacity portion of nanoparticles $\left( {\phi_{1} } \right)$. Here are trampled lines for hybrid nanofluid *Ag* + *COFe*_*2*_*O*_*4*_ and dash lines for nanofluid *COFe*_*2*_*O*_*4*_. Figure 3 designates the demonstration of the density segment of nanoparticles $\left( {\phi_{2} } \right)$ against the velocity distribution profile $F\left( \zeta \right)$. The velocity profile $F\left( \zeta \right)$ is condensed by the advanced extent of the volume fraction of nanoparticles $\left( {\phi_{2} } \right)$. The inspiration for the velocity distribution profile $F\left( \zeta \right)$ in terms of the magnetic parameter $\left( M \right)$ is indicated in Fig. 4. The boosting variation of the magnetic parameter $\left( M \right)$ falls in the velocity distribution profile $F\left( \zeta \right)$. This is owing to the magnetic force that affected the fluid motion, a resistant force is produced which hampers the movement of the fluid. Here are solid lines for hybrid nanofluid Ag + COFe_2_O_4_ and dashes lines for nanofluid COFe_2_O_4_. The performance of the magnetic parameter $\left( M \right)$ over the velocity distribution profile $G\left( \zeta \right)$ of fluid is detected in Fig. 5. From the figure, it can be seen that the swiftness distribution profile $G\left( \zeta \right)$ has decreasing behavior for the higher magnetic parameter $\left( M \right)$. A magnetic field is a vector field that explains the magnetic impact on electric currents, current flow, and magnetic fluids. A flowing charge in a magnetic field is subjected to a force that is perpendicular to both its velocity and the magnetic field. Figure 6 defines the presentation of the volume segment of nanoparticles $\left( {\phi_{1} } \right)$ versus the momentum distribution profile $G\left( \zeta \right)$. The velocity distribution profile $G\left( \zeta \right)$ is reduced by the increased intensity of the volume segment of nanoparticles $\left( {\phi_{1} } \right)$. Here are compacted lines for hybrid nanofluid *Ag* + *COFe*_*2*_*O*_*4*_ and dash outlines for nanofluid *COFe*_*2*_*O*_*4*_. Figure 7 illustrates the power concentration of nanoparticles $\left( m \right)$ on the velocity distribution profile $G\left( \zeta \right)$. The cumulative principles of concentration of nanoparticles $\left( m \right)$ increased the swiftness distribution profile $G\left( \zeta \right)$. The concentration of nanoparticles $\left( m \right)$ is directly associated with the velocity of the fluid. Here are solid lines for hybrid nanofluid *Ag* + *COFe*_*2*_*O*_*4*_ and dashes lines for nanofluid *COFe*_*2*_*O*_*4*_. The salient features of the volume segment of nanoparticles $\left( {\phi_{2} } \right)$ on the high-temperature distribution profile $\theta \left( \zeta \right)$ are deliberated in Fig. 8. The lines of graphs display that the rising reliabilities of the volume segment of nanoparticles $\left( {\phi_{2} } \right)$ increased the temperature distribution profile $\theta \left( \zeta \right)$. The features of the volume segment of nanoparticles $\left( {\phi_{1} } \right)$ via the temperature dispersal profile $\theta \left( \zeta \right)$ are pictured in Fig. 9. The temperature dissemination profile $\theta \left( \zeta \right)$ is enhanced by the intensifying ethics of the volume segment of nanoparticles $\left( {\phi_{1} } \right)$. Here comparative study is investigated and solid lines for hybrid nanofluid Ag + COFe_2_O_4_ and dashes lines for nanofluid COFe_2_O_4_.

Table 1 shows the thermophysical properties of nanofluid. Table 2 analyzed the thermophysical properties of a hybrid nanofluid. Table 3 shows the importance of the thermophysical properties of nanoparticles including such (*Ag*) and (*COFe*_*2*_*O*_*4*_) with base fluid Water. Table 4 displayed the shape factor of particles like a sphere, hexahedrons, tetrahedrons, cylinders, columns, and lamina. Table 5 shows the variation of magnetic parameters via the Nusselt number. It shows good agreement between published and current work. Table 6 analyzed the comparison of *Nu* results at disk and cone with those of previous research utilizing just the common parameters. It analyzed the best agreement between both results published and current work.

**Table 1:** Properties of nanofluid^43^.

Properties	Formula
Density $\left( {\rho_{nf} } \right)$	$\rho_{nf} \left( { = \phi_{1} \left( {\rho_{{s_{1} }} } \right) + \rho_{f} \left( {1 - \phi_{1} } \right)} \right)$
Viscosity $\left( {\mu_{nf} } \right)$	$\mu_{nf} \left( { = \mu_{f} /\left( {1 - \phi_{1} } \right)^{2.5} } \right)$
Heat capacity $\left( {\rho C_{p} } \right)_{nf}$	$\left( {\rho C_{p} } \right)_{nf} \left( { = \phi_{1} \,\left( {\rho C_{p} } \right)_{{s_{1} }} + \left( {\rho C_{p} } \right)_{f} \left( {1 - \phi_{1} } \right)} \right)$
Thermal conductivity $\left( {k_{nf} } \right)$	$\frac{{k_{nf} }}{{k_{f} }}\left( { = 2k_{f} + k_{{s_{1} }} - 2\left( {k_{f} - k_{{s_{1} }} } \right)\phi_{1} /\phi_{1} \left( {k_{f} - k_{{s_{1} }} } \right) + k_{{s_{1} }} + 2k_{f} } \right)$

**Table 2:** Properties of hybrid nanofluid^43^.

Properties	Formula
Density $\left( {\rho_{hnf} } \right)$	$\rho_{hnf} \left( { = \phi_{2} \rho_{s2} + \left( {1 - \phi_{2} } \right)\left( {\left( {1 - \phi_{1} } \right)\rho_{f} + \phi_{1} \rho_{s1} } \right)} \right)$
Viscosity $\left( {\mu_{hnf} } \right)$	$\mu_{hnf} \left( { = \mu_{f} /\left( {1 - \phi_{2} } \right)^{2.5} \left( {1 - \phi_{1} } \right)^{2.5} } \right)$
Heat capacity $\left( {\rho C_{p} } \right)_{hnf}$	$$\left( {\rho C_{p} } \right)_{hnf} \left( { = \phi_{2} \left( {\rho C_{p} } \right)_{s2} + \left( {1 - \phi_{2} } \right)\left( \begin{gathered} \left( {1 - \phi_{1} } \right)\left( {\rho C_{p} } \right)_{f} \hfill \\ + \phi_{1} \left( {\rho C_{p} } \right)_{s1} \hfill \\ \end{gathered} \right)} \right)$$
Thermal conductivity $\left( {k_{hnf} } \right)$	$$\begin{gathered} \frac{{k_{hnf} }}{{k_{nf} }}\left( { = k_{s2} + 2k_{hnf} - 2\left( {k_{hnf} - k_{s2} } \right)\phi_{2} /k_{s2} + 2k_{hnf} + \phi_{2} \left( {k_{hnf} - k_{s2} } \right)} \right), \hfill \\ Here\, \hfill \\ \frac{{k_{hnf} }}{{k_{nf} }}\left( { = k_{s1} + 2k_{f} - 2\left( {k_{f} - k_{s1} } \right)\phi_{1} /k_{s1} + 2k_{f} + \phi_{1} \left( {k_{f} - k_{s1} } \right)} \right) \hfill \\ \end{gathered}$$

**Table 3:** Nanoparticle's thermophysical properties^44,45^.

**Particles**	$\rho$ **Density**	$Cp$ **Heat capacity**	$k$ **Thermal conductivity**
	4907	700	3.7
	10,500	230	418
	997.1	4179	0.613

**Table 4:** Geometrical appearance and shape factor^46,47^.

Shape name	Shape factor
	3.0
	3.7
	4.0
	4.9
	6.3
	16.1

**Table 5:** Variation of magnetic parameter via Nusselt number^48,49^.

$\left( M \right)$	Moatimid et al.^48^	Rooman et al.^49^	Current work
0.0	1.1175	1.1175289	1.1175293
0.1	1.114	1.1103288	1.1103290
0.2	1.1105	1.1021636	1.1021644
0.3	1.107	1.0928879	1.0928889
0.4	1.1035	1.0823038	1.0823037
0.5	1.1	1.0702154	1.0702159

**Table 6:** Comparison of Nusselt number results for disk and cone with those of previous research^41,42^ utilizing just the common parameters.

*n*	$\theta^{\prime}\left( 0 \right)$			$\theta^{\prime}\left( 1 \right)$		
	Turkyilmazoglu^41^	Gul et al.^42^	Current work	Turkyilmazoglu^41^	Gul et al.^42^	Current work
1	0.757932	0.758845	0.7590311	1.420219	1.421320	1.422432
2	0.766821	0.767734	0.7681200	1.431320	1.432431	1.433541
3	0.775701	0.776623	0.7770101	1.442431	1.443542	1.444654

## Graphical plots

See Figs. 2, 3, 4, 5, 6, 7, 8, 9 and Tables 4, 5, 6.

## Final observations

In the current work, real uses of hybrid nano liquid containing $$Ag + COFe_{2} O_{4}$$$$COFe_{2} O_{4}$$ nanoparticles and water as a based fluid are measured against velocity and temperature profiles. The main consequences are as follows:

- The velocity distributions profiles are reduced for the superior estimations of the volume segment of nanoparticles $\phi_{1}$ and volume segment of nanoparticles $\phi_{2}$
- The velocity distribution profile is decreased for the higher magnitude of the magnetic parameter $M$. Here comparative study is investigated and reliable lines for hybrid nanofluid $Ag - COFe_{2} O_{4}$ and dashes lines for nanofluid $COFe_{2} O_{4}$.
- The temperature distributions profile is boosted up for the greater values of the volume segment of nanoparticles $\phi_{1}$
- The temperature distributions profile is boomed for the greater values of volume fraction of nanoparticles $\phi_{2}$

## Data Availability

All data generated or analyzed during this study are included in this manuscript.
